# Unsafe abortion in rural Tanzania – the use of traditional medicine from a patient and a provider perspective

**DOI:** 10.1186/s12884-014-0419-6

**Published:** 2014-12-19

**Authors:** Vibeke Rasch, Pernille H Sørensen, Anna R Wang, Flora Tibazarwa, Anna K Jäger

**Affiliations:** Department of Obstetrics and Gynaecology, Odense University Hospital, 5000 Odense C, Denmark; Department of International Health, Immunology and Microbiology, Faculty of Health and Medical Sciences, University of Copenhagen, 3D Blegdamsvej, 2200 Copenhagen N, Denmark; The Medical Faculty, University of Southern Denmark, 5000 Odense C, Denmark; Department of Botany, University of Dar es Salaam, Dar es Salaam, P.O. Box 35060, Tanzania; Department of Drug Design and Pharmacology, Faculty of Health and Medical Sciences, University of Copenhagen, 2 Universitetsparken, 2100 Copenhagen O, Denmark

**Keywords:** Unsafe abortion, Provider, Method, Traditional, Plants, Tanzania

## Abstract

**Background:**

The circumstances under which women obtain unsafe abortion vary and depend on the traditional methods known and the type of providers present. In rural Tanzania women often resort to traditional providers who use plant species as abortion remedies. Little is known about how these plants are used and their potential effect.

**Methods:**

Data were obtained among women admitted with incomplete abortion at Kagera Regional Hospital during the period January - June, 2006. The women underwent an empathetic interview to determine if they had experienced an unsafe abortion prior to their admission. In all 125/187 women revealed having had an unsafe abortion. The women identified as having had an unsafe abortion underwent a questionnaire interview where information about abortion provider and abortion method used was obtained through open-ended questions. To get more detailed information about the traditional methods used to induce abortion, in-depths interviews and focus group discussions were performed among traditional providers and nurses. Finally, the plant specimen’s effectiveness as abortion remedies was assessed through pharmacological analyses.

**Results:**

Among women admitted with incomplete abortions, 67% had had an unsafe abortion. Almost half of the women who had experienced an unsafe abortion had resorted to traditional providers and plant species were in these cases often used as abortion remedies. In all 21 plant species were identified as potential abortion remedies and analysed, 16 of the species were found to have a uterine contractive effect; they significantly increased the force of contraction, increased the frequency of contractions or did both.

**Conclusion:**

Unsafe abortion is common in rural Tanzania where many women use plant species to terminate an unwanted pregnancy. The plants have a remarkable strong uterine contractive effect. To further understand the consequences of unsafe abortion there is a need for further analyses of the plants’ potential toxicity and mutagenicity.

## Background

Globally, 40% of women of childbearing age (15–44) live in countries with highly restrictive laws where abortion is prohibited altogether or only allowed to save a woman’s life or protect her physical or mental health [1]. In countries where abortion is illegal, women have to resort to illegal and often unsafe interventions to get an unwanted pregnancy terminated. In most parts of the world affluent women have virtually always been able to obtain safe abortions, performed by a skilled provider under hygienic circumstances, regardless of law codes or religious decrees. In contrast, women who cannot afford to pay a skilled provider often have to resort to unsafe methods. Unsafe abortion is a significant contributor to the high maternal mortality rate in low income countries and accounts for 13 percent of all maternal deaths [2].

The circumstances under which women obtain unsafe abortion varies from one setting to another and depends on the traditional methods known, the type of providers present and the availability of trained health professionals who are willing to perform abortion despite the intervention being illegal. In a recent study from Pakistan, 80% of the women seeking abortion had terminations performed by health professionals, either doctors, lady health visitors or nurses [3]. A similar picture has been found in Burkina Faso, where 61% of all abortions were induced by a health professional [4]. Other commonly used providers are traditional healers and traditional birth attendants as documented in a study from Guatemala where 49% of abortions had been induced by a traditional provider [1]. Similarly, a recent study from Tanzania showed that almost half of the women, who were admitted with abortion complications after an unsafely performed induced abortion, had resorted to a traditional provider who had attempted to induce the abortion by use of traditional methods whereas the other half of the women had resorted to a medical professional [5]. Most of the documented traditional methods consist of different plant specimens, which are brewed as tea alone or in combination with one another [5,6]. Occasionally the plant specimens may also be administered vaginally [5,7].

Women in most cultures have throughout history employed traditional methods to control their fertility and anthropologists studying contraception and abortion in non-western societies have reported the use of an enormous number of plant specimens, but they have rarely focused on the substances’ effects and side effects [8]. Additionally, most medical professionals and scientist have considered traditional use of plant specimens as merely symbolic or mythic with no physiological foundation. Therefor the information, which is available about how traditional providers induce abortion, is mostly of anecdotal character provided by women who have been admitted with abortion complications or health staff who have attended women with abortion. Hence there is a lack of detailed, structured information, which describes the remedies and methods used to induce abortion in more detail. We have previously through a quantitative approach described that unsafe abortion is a significant problem in hospital based settings in Tanzania [9], we have also described that many of the women who are admitted with abortion complications have had their abortion induced by traditional providers who are using roots and plant specimens to induce abortion [5] and we have documented that some of the herbs used to induce abortion have a significant uterus contractive effect [10]. Many women who experience an unwanted pregnancy resort to traditional medicine, either provided by a traditional provider, a family member or the woman/girl herself [5]. However detailed information about how these herbs are being used is lacking. Hence to better understand the problem of induced abortion and the associated risk, there is a need of more detailed information on how plant specimens are used to induce abortion. In this paper we therefore explore the use of traditional medicine from a patient and a provider perspective and describe in detail how the plant specimens are prepared and administered.

## Method

### Study setting

The study was conducted in Kagera Region in North Western Tanzania. Kagera Region comprises six districts: Karagwe, Ngara, Biharamulo, Muleba, Bukoba Rural and Bukoba Urban. It has a regional hospital, Kagera Regional Hospital, which functions as a first line hospital for the people living in Bukoba Urban and as a second line hospital for the people in Kagera Region. Data were collected at Kagera Regional Hospital and in Bukoba Rural district.

### Material

To get a detailed understanding of how women who experience an unwanted pregnancy in rural Tanzania make use of traditional providers to interrupt their pregnancy, a combined quantitative and qualitative approach was utilised together with an ethno-pharmacological approach. This mix of methods, which are often referred to as triangulation, enabled us to describe how women make use of traditional providers and plant specimens when aiming at having an unwanted pregnancy terminated, how traditional providers use plant specimens to induce abortion and which plant specimens they perceive as effective and finally whether these plant specimens have a significant uterus contractive effect and thus may be considered as potential abortion remedies. More specifically, women admitted with complications after unsafe abortion underwent a face to face interview which included open-ended questions about how the abortion was performed. In-depth interviews and focus group discussions were additionally performed among traditional providers and nurses and finally plant species were collected and a pharmacological study was performed.

#### Questionnaire interviews and case notes

A total of 187/223 (84%) women admitted with incomplete abortion in Kagera Regional Hospital during the period January – June 2006 were included in the study. By the use of a previously described empathetic approach, the women were classified as having had either an unsafe abortion or a spontaneous abortion [5]. This approach is based on a private, confidential in-depth dialogue performed without any questionnaire or notes. When the interviewer feels she has gotten trustworthy information about the abortion situation and the circumstances characterising it, the informant is asked permission to record the obtained information in a structured questionnaire. Of the 187 women admitted with an incomplete abortion 125 revealed having had an unsafely induced abortion prior to admittance in the hospital. All women who were identified as having had an unsafe abortion were asked to describe the circumstances characterising the induction in detail. The information obtained about abortion provider and abortion method was written down as case stories to describe the abortion experience from the women’s point of view.

#### In-depth interviews and focus group discussions

In all 21 traditional providers residing in Bukoba Rural district and 2 nurses participated in in-depth interviews. The traditional providers were identified via snowball sampling, where we first identified three traditional providers and interviewed them and thereafter asked them to provide information needed to locate other traditional providers in the district. The interviews focused on plant species used in relation to pregnancy and delivery complications, plant specimens perceived as effective abortion remedies, how the plant specimens were prepared and used. Data saturation was used as a guiding principle and after having interviewed 21 traditional birth attendants it was felt that the information obtained was repeating what was already found in the previous interviews and that further interviews would not add to the findings. The interviews were conducted by VR and PHS together with a Tanzanian nurse/midwife with extensive experience in conducting interviews among women having experienced unsafe abortion. In addition, two focus group discussions were organized, one involving three traditional providers, who had participated in the in-depth interviews and were willing to participate in a focus group discussion and another one involving four nurses, who had knowledge about traditional medicine used in the area. During the focus group discussions, special emphasis was placed on plant species used to induce abortion, their potential effect as abortion-inducing remedies and how they were prepared and used.

#### Plant specimens

Based on the information provided by the traditional providers and nurses, 98 different plants which were used for pregnancy and delivery complications were identified. Sixty-four of these plants were assumed to have a uterine contractive effect and through discussions with the traditional providers and the nurses, 21 plants used commonly for induced abortion were selected for further analysis. Plant material was collected during two periods, January – June 2006 and October-November 2008. The local names, collection dates, site and coordinates were recorded. The plants were authenticated at the Institute of Botany, Dar es Salaam, Tanzania and voucher specimens are deposited there. The plant material was dried and stored in brown paper bags. They were subsequently brought to University of Copenhagen for extraction and further analyse.

### Data analyses

Questionnaire data were entered in Epi Info version 6.04 from Epidemiology and Disease surveillance, CDC, Atlanta, USA. One way frequency tabulations were performed to summarize the data obtained on abortion method, abortion provider and place of abortion.

The in-depth interviews were tape recorded if the interviewee accepted and subsequently transcribed. If the interviewee refused to be tape recorded, the interviews were recorded manually by the research assistant who took extensive notes during the interviews and wrote as many quotations down as possible. Template analysis style was applied as a systematic procedure to organise, compare and validate alternative interpretations of the data [11]. The starting point for the construction of the initial template was the interview topic guideline. After the initial template was constructed, the full set of transcripts and notes was read systematically and themes in the text were identified and grouped together into the thematic categories e.g. plant specimens used for reproductive health, plants specimens used for abortion, preparation of plant specimens and instruction to the girl/woman.

Extracts of the plant species used for preparation of abortion-inducing remedies were tested for uterus contraction. This part of the study has previously been described in detail [10]. In brief, an organ-based assay utilising uterine tissue from an oestrus rat was used to test for uterine contraction. The uterine horn of the rat was isolated and mounted in an organ bath containing 1.0 ml De Jalon solution (NaCl 154 mM; KCl 5.6 mM; NaHCO_3_ 17.9 mM; CaCl_2_ 2.2 mM; glucose 2.5 mM). The bath was continually aerated with 5% CO_2_ in O_2_ and maintained at 30°C to minimize spontaneous contraction. 1 g of each plant was grounded and extracted with 20 ml ethanol and the extracts were subsequently tested by cumulatively addition, with the following concentrations in the organ bath: 0.04; 0.14; 0.44 and 1.40 mg/ml. Additions were done with 6 min intervals. Before testing of a new extract the organ bath was flushed several times and a test with acetylcholine performed.

### Ethics

All study participants were informed that participation was voluntary. Oral informed consent was obtained since most of the women who had experienced an unsafely induced abortion as well as the traditional providers feared lack of anonymity if they signed a written form of consent. The study, including the approach to obtain informed consent, was ethically approved by the National Institute of Medical Research in Dar es Salaam, Tanzania.

## Results

### Experiences from women having unsafe abortion

Among the women admitted with incomplete abortion, 67% (125/187) revealed having had an unsafe abortion and 45% of these women had resorted to an unskilled provider, either a traditional provider or a relative, to get their unwanted pregnancy terminated (Table 1).

**Table 1:** **Abortion method, abortion provider and place of abortion**

	**N =125**	**%**
Method		
MVA/D&C	46	38.0
Catheter	16	13.2
Roots	10	8.3
Plant species, vaginal	16	13.2
Plant species, oral	26	21.5
Other	7	5.8
Missing	4	
Provider		
Patient/family	33	27.0
Traditional healer	22	18.0
Midlevel provider^a^	50	41.0
Doctor^b^	17	13.9
Missing	3	
Place		
Public hospital	19	15.7
Private hospital	21	17.4
Health center	13	10.7
Private house	68	56.2
Missing	4	

^a^Nursemidwife, clinical officer.

^b^Assistant medical officer, medical officer.

Plant species used orally or intra-vaginally had been used to induce the abortion among 22% and 13%, respectively. Another 8% of the women stated that the abortion had been induced by a cassava stem which was used mechanically to rupture the membranes.

The case notes provided a more detailed picture of how the plant species were used. The most common way of using the herbs was to take them orally, most often consumed as a strong tea or chewed, and the women often combined different types of herbs in their attempts to interrupt the pregnancy: *The woman was instructed in drinking a strong tea of akashwagara together with barks of lemon roots. Since contraction had not yet occurred the following day, she chewed “eiyabuya” and afterwards she started to have contraction and bleeding. The foetus was expelled but placenta retained and the woman went to a nearby health clinic and was subsequently referred to the hospital for treatment (idnr 67).*

The herbs were frequently used intra-vaginally, in some situations the plant species were pounded and placed in the vagina by the traditional provider: *Went to a traditional healer who pounded some local herbs and inserted them into the vagina using gloved fingers. The herbs were removed and the woman was instructed to go home and wait for expulsion and report to a health facility when she experienced heavy bleeding* (idnr 148). In other situations, the woman had prepared the herbs herself and inserted them in the vagina: *The woman powdered “eibezi” and put it into the reproductive tract. Then she started feeling abdominal pain and bleeding per vagina (slight). Decided to come to hospital for help* (idnr 148).

Many women had also visited a traditional provider who used roots to induce the abortion: *A herbal stick was introduced into cervix and left there to open the cervix and thereby interfere with foetal life – the stick was removed when bleeding started and the girl later reported at the hospital for treatment”* (idnr 129). Or they had inserted the stick them self with the help from a relative/friend: *A friend assisted her in inserting a cassawa leaf-stalk into the vagina. It was left in position and was supposed to be removed after she felt contractions, even if she didn’t get strong contractions. As the procedure would have to be repeated after 3 days* (idnr 130).

In many situations, the women had been advised to attend the hospital for further treatment when bleeding occurred: *The woman was given herbs to chew and others put into birth canal and told to take them off when contractions and bleeding start. Then go to hospital for more assistance* (idnr 57).

### Experiences from traditional providers and nurses

More detailed information about the types of plant species used and how they were used were provided through in depths interviews with the traditional providers and nurses. Twenty one plant species were identified as commonly being used as abortion-inducing remedies (Table 2). According to the interviewees, most of the plants were taken orally, often in large amounts as a concentrated brew or alternatively chewed. A few of the plant species were used intra-vaginally. Eyabya was one of the most commonly used plants and mentioned by many of the traditional providers and nurses:

**Table 2:** **Plant species used for induction of abortion in Tanzania**

	**Local name**	**Latin name**	**Effect on force of contraction**	**Effect on frequency of contraction**
1	Akakurura	*Bidens pilosa* L.	++	0
2	Akaramata	*Rubia cordifolia*	+	+
3	Akashwagara	*Ocimum suave* Klilld.	++	0
4	Eitezi	*Commelina africana* L.	++	+
5	Ekijumbura	*Obetia radula* (Baker) B.D.Jacks.	+	+
6	Engenyi	*Zehneria scabra* (Linn. F.) Sond.E	0	+
7	Enkaka Aloe Vera	*Aloe sp*	0	+
8		*Oldenlandia corymbosa* L.	++	0
9	Eyabia	*Macrotyloma axillare* (E.Mey.) Verdc	0	0
10	Eyabya no. 2	*Manihot esculenta* Crantz	++	0
11	Kaitampunu	*Triumfetta microphylla* Wight & Arn	+	+
12	Kikikarabo	*Azadirachta indica* A. Juss.	0	+
13	Muarobaini	*Desmodium barbatum* (L.) Benth	++	+
14	Omubabazi no. 1	*Sphaerogyne latilifolia* Naudin	0	0
15	Omubabazi no.2	*Vernonia amygdalina* Delile	++	+
16	Omubirizi	*Citrus sinensis* (L.) Osbeck	0	0
17	Omudimu	*Ricinus communis* L.	+	0
18	Omujuna	*Canthium* sp.	0	+
19	Omushangati	*Ficus thonningii* Blume	0	0
20	Omutoma	*Cassia mimosoides* L.	0	0
21	Orwangwe	*Biophytum helenae* Buscal & Muschi	0	+
	Webumbe			

+ The extract induced contractions of more than 50% of the maximum contraction obtained with acetylcholine after adjustment for spontaneous contractions in at least one test concentration.

++ The extract induced contractions of the same strength as the maximum response obtained with acetylcholine in at least one test concentration.

*“Eyabya is known by many and is often used to induce abortion. Since it grows everywhere and is easy to find…… The leaves are powdered and mixed with cold water to a juice. The woman will have to drink 500 ml 3 times a day. She will have to repeat this each day until she gets contractions or starts bleeding. Normally it takes 3–7 days before the effect appears.”*

[PHNurse1]

The other plants mentioned were in general used in the same way. The criteria for using a specific plant was that it was easily available for the traditional providers, who often had a selection of different plants, growing in their “shambas”:

*“You can use the leaves from Kaitampunu - a type of cassava - to induce abortion. The leaves are squeezed and mixed with water. The woman has to drink a large dose first and then continue with smaller doses. The contractions will normally start after about 12 hours……. The plant can be found nearby and there is no risks using it. When the woman begins to bleed she has to drink a mixture made of the juice from the leaves of Ekinami and cold water to stop the bleeding.”*

[PHTP1]

Another way to increase the chance of a successful outcome was to add other plant species to the treatment if the initially used plants were found to be ineffective:

*“Ekijumbura is used to increase contractions. The plant species (leaves or roots) are pounded, chewed and swallowed. After hours the foetus will be expelled…… The women are thereafter given Omeshasha to stop the bleeding. If Ekijumbura fails, Engenyi roots may be used… Ekijumbura is used first since it is easy to find. The woman is first asked to chew a piece of Ekijumbura root and while she is chewing the root I will prepare a tea [utilising the same plant specimen] and go looking for the Engenyi roots which I will use if Ekijumbura fails.”*

[VRTP4]

### Pharmacological examination

Extracts of the 21 plant species used for preparation of abortion-inducing remedies were tested for their uterine contractive effect (Table 2). Both the force and frequency of the contractions were recorded. In all 16 of the 21 collected plant species were found to have a uterine contractive effect; they either increased the force of contraction, increased the frequency of contractions or did both [10]. The species that gave the strongest contractions comparable to the maximum response obtained with acetylcholine, a well-known uterine contracting agent, were *Bidens pilosa*, *Commelina africana, Desmodium barbatum, Manihot esculenta, Ocimum suave* and *Sphaerogyne latifolia.* Extracts of seven species, *Commelina africana*, *Desmodium barbatum, Obetia radula, Ocimum suave, Rubia cordifolia, Sphaerogyne latifolia* and *Triumfetta microphylla* increased both the force of contractions and their frequency. For most of the extracts, a dose–response curve was observed.

## Discussion

More than two-third of the women who were admitted with an incomplete abortion reported having had an unsafely induced abortion and one third of these women had used a plant specimen as abortion remedy. The plants used were found to have a remarkable strong uterine contractive effect.

Pregnancy termination is only legally available in Tanzania if the pregnancy is a threat to the woman’s life. According to the law, any person who provides illegally induced abortion risks 14 years imprisonment and any person who provides remedies to induce abortions are at risk of three years imprisonment, whereas the woman herself risks seven years imprisonment. The legal context of abortion makes it a great challenge studying the problem of unsafe abortion and naturally any researcher focusing on the problem have to work with great diligence. Reliable data on abortion are difficult to obtain, both due to the illegality of the procedure as well as the social stigma associated with it. However, it is believed that the empathetic approach utilised [9] enabled us to identify women having experienced an unsafe induced abortion. It is also important to stress that many of the traditional providers interviewed were reluctant to be tape recorded for fear of the legal consequences, and it should be kept in mind that the traditional providers who gave us the information about the different plants used to induce abortion, might not themselves have performed the procedure, but they knew about it and in case they did not provide the plant species themselves they could pass the instructions on to the women in need.

More than two-third of the women who were admitted with an incomplete abortion reported having had an unsafely induced abortion prior to admittance in the hospital. Similarly high rates of unsafe abortions have been reported in other Tanzanian studies [9,12,13]. Almost half of the women had either turned to traditional providers to have a clandestine abortion or self-induced their abortion. This finding is in line with other studies documenting that rural women often resort to traditional providers if they want to have an unwanted pregnancy terminated [1,7]. Traditional providers often play a major role in helping poor and rural families meet healthcare needs of all kinds due to their familiarity, accessibility and affordability. In addition most rural women often do not have the means to cover transport to a health clinic or hospital and/or the means to cover the cost of having an abortion performed by a skilled provider. Therefore they may be more prone to resort to traditional providers in comparison with urban women [1].

From the case notes, it was apparent that many women/girls had induced the abortion themselves by using traditional medicine. In addition, the study revealed that many of the plants used to induce abortion are easily accessible as they grow in people’s shambas, along the roadsides and in other places where people can easily access them. Hence many women know of plant specimens considered effective as abortion remedies and also know where to find them. Consequently they often are in a position where they can attempt to induce an abortion without involving a traditional provider or a health professional.

There is generally a concern about how effective and dangerous plant specimens are when used as abortifaciants [1]. So far only very few studies have documented their potential effect not to mention possible side effects. In the present study the uterine contractive effect of the plants were examined pharmacologically through a previously described uterine assay [10]. Other studies have also used this method successfully to test plant species’ uterine contracting effect [14-20]. In all 16 of the 21 collected plant species were found to have a uterine contractive effect, they either increased the force of contractions, the frequency of contractions or both. Some of the collected plant species have previously been tested for their potential use as abortion remedies. *Bidens pilosa* has been tested in a uterus assay where the extract elicited strong uterine contractions [20] and an extract from the seeds of *Ricinus communis* has also previously been documented to be a strongly abortifacient [21,22]. Likewise analyses of *Azadirachta indica* have shown an abortifacient effect in both rats and primates [23]. Based on the findings from the present study and other studies it may be argued that most of the plant species used for abortion in the setting studied are potentially effective abortion remedies. The potential harmful effects of the different plant species have not yet been described thoroughly and there is a need for thorough examination of plant extracts to test them for potential toxicity and mutagenicity. This concern is supported by a study from Montevideo Poison Centre that examined the toxic effect of herbs used with abortive intent, in 5 out of 86 cases deaths had occurred following the herbal ingestion [24]. Further in a Tanzanian study, traditional providers revealed that dosages of plant medicine were not specific and that their side effects were not known [25].

Empirical studies on the safety of abortions that are illegal are rare. It is assumed that pregnancy terminations are safer if they are performed by a qualified doctor than if performed by lay health workers, unskilled practitioners or traditional providers [1]. However, a study from Burkina Faso has documented that abortions induced by traditional providers and health workers have about the same “complication” rate [4]. Likewise two recent studies from Nigeria and Tanzania, which compared the complication rates between different abortion methods, found that the method most often associated with abortion complications was catheter/roots whereas plant species was the method associated with least complications [5,26]. These findings are in line with the common notion that the more invasive the technique is, the more dangerous it is to the woman [27]. Apparently the reality of the safety of clandestine abortion interventions is more complex than generally believed.

There are some noteworthy limitations of this study. Firstly, in the present study we managed to identify women who had attempted to terminate an unwanted pregnancy by using traditional medicine, however, these women only represent woman who were admitted to the hospital because of abortion complications. Hence, the study does not cover women who were using traditional medicine to induce an abortion without experiencing any complications. Secondly, to identify traditional providers, who helped the women in terminating an unwanted pregnancy, snowball sampling was used. Through this approach we managed to identify 21 traditional providers who were willing to share their experience with us. However, snowball sampling is hardly likely to lead to a representative sample and the results from this study cannot be extrapolated to other areas of Tanzania where other types of herbs may be used and where the herbs also may be used in a different way. Thirdly, the herbs we collected were only tested for their uterine contractive effect. Some of the herbs as e.g. *Eyabya,* which often were mentioned by the traditional providers as an effective abortifaciant, did not have any contractive effect. This should not be interpreted as Eyabya not being an effective abortifaciant, it could easily act via another mechanism which the present study design did not cover. Perhaps even more important, the study did not allow us to investigate possible toxic effect of the different herbs. Hence, it has to be underlined that the present study only provides a small piece of information in a complex picture of how traditional medicine can be used to induce abortion and there is a great need of more studies on pharmacokinetics, pharmacodynamics and possible toxicity to get a better understanding of the whole picture of unsafe abortion in rural Tanzania.

## Conclusion

Unsafe abortion is common in rural Tanzania where many women and girls either resort to a traditional provider or self-induce an abortion to terminate an unwanted pregnancy. The plants used for induction have a remarkable strong uterine contractive effect, however the potential harmful effects of the different plant species have not yet been described thoroughly and there is a need for thorough examination of plant extracts to test them for potential toxicity and mutagenicity.
