# Erratum: Unsafe abortion in rural Tanzania – the use of traditional medicine from a patient and a provider perspective

**DOI:** 10.1186/s12884-015-0794-7

**Published:** 2015-12-29

**Authors:** Vibeke Rasch, Pernille H. Sørensen, Anna R. Wang, Flora Tibazarwa, Anna K. Jäger

**Affiliations:** Department of Obstetrics and Gynaecology, Odense University Hospital, Odense C, 5000 Denmark; Department of International Health, Immunology and Microbiology, Faculty of Health and Medical Sciences, University of Copenhagen, 3D Blegdamsvej, Copenhagen N, 2200 Denmark; The Medical Faculty, University of Southern Denmark, Odense C, 5000 Denmark; Department of Botany, University of Dar es Salaam, Dar es Salaam, Tanzania; Department of Drug Design and Pharmacology, Faculty of Health and Medical Sciences, University of Copenhagen, 2 Universitetsparken, Copenhagen O, 2100 Denmark

## Erratum

The original version of this article [1] unfortunately contained a mistake. The presentation of Table 2 was incorrect in the HTML and PDF versions of this article with the following errors:

**Table 2:** Plant species used for induction of abortion in Tanzania

	Local name	Latin name	Effect on force of contraction	Effect on frequency of contraction
1	Akakurura	*Bidens pilosa* L.	++	0
2	Akaramata	*Rubia cordifolia*	+	+
3	Akashwagara	*Ocimum suave* Klilld.	++	+
4	Eitezi	*Commelina africana* L.	++	+
5	Ekijumbura	*Obetia radula* (Baker) B.D.Jacks.	+	+
6	Engenyi	*Zehneria scabra* (Linn. F.) Sond.E	0	+
7	Enkaka Aloe Vera	*Aloe sp*	0	+
8	Eyabia	*Oldenlandia corymbosa* L.	++	0
9	Eyabya no. 2	*Macrotyloma axillare* (E.Mey.) Verdc	0	0
10	Kaitampunu	*Manihot esculenta* Crantz	++	0
11	Kikikarabo	*Triumfetta microphylla* Wight & Arn	+	+
12	Muarobaini	*Azadirachta indica* A. Juss.	0	+
13	Omubabazi no. 1	*Desmodium barbatum* (L.) Benth	++	+
14	Omubabazi no.2	*Sphaerogyne latilifolia* Naudin	++	+
15	Omubirizi	*Vernonia amygdalina* Delile	0	0
16	Omudimu	*Citrus sinensis* (L.) Osbeck	0	0
17	Omujuna	*Ricinus communis* L.	+	0
18	Omushangati	*Canthium* sp.	0	+
19	Omutoma	*Ficus thonningii* Blume	0	0
20	Orwangwe	*Cassia mimosoides* L.	0	0
21	Webumbe	*Biophytum helenae* Buscal & Muschi	0	+

The local name ‘Eyabia’ has moved down a row causing subsequent rows to move out of order.

In addition, there are several errors in the reported effects of *Ocimum suave*, *Vernonia amygdalina* and *Sphaerogyne latifolia*.

The corrected Table 2 is given below.
